# Assessment of Immune Cell Populations in the Peripheral Blood of Patients With Metastatic Prostate Cancer

**DOI:** 10.7759/cureus.80672

**Published:** 2025-03-16

**Authors:** Vanessa Patel, Patrícia Corredeira, Ana Cavaco, Tiago Barroso, Pedro Filipe, André Mansinho, Catarina Abreu, Lisiana Wachholz Szeneszi, Julie Ribot, Bruno Silva Santos, Luís Costa

**Affiliations:** 1 Oncology, Unidade Local de Saúde Santa Maria, Lisbon, PRT; 2 Laboratory, Gulbenkian Institute for Molecular Medicine, Lisbon, PRT; 3 Medicine and Biomedical Sciences, Algarve University, Faro, PRT; 4 Research, Gulbenkian Institute for Molecular Medicine, Lisbon, PRT

**Keywords:** metastatic prostate cancer, oligometastatic disease, peripheral immune cell, plurimetastatic disease, γδ2+ t cells

## Abstract

Background

Prostate cancer (PCa) encompasses a heterogeneous spectrum, ranging from indolent to highly aggressive forms, with approximately 10-20% of patients with initially localized disease later becoming metastatic. Oligometastatic PCa (OMPC) represents an intermediate state between locally advanced and high-volume metastatic disease. Understanding the immune landscape of OMPC and plurimetastatic PCa (PMPC) can provide valuable insights into disease biology, with potential implications for treatment strategies and prognosis.

Aim and objective

This study aimed to evaluate alterations in circulating immune cell subsets between OMPC and PMPC to identify potential immune biomarkers and therapeutic targets.

Methods

We conducted a retrospective cohort study of 43 mPC patients. Patients were stratified into two groups based on metastatic spread: OMPC (≤5 metastatic lesions in bone or lymph nodes) and PMPC (>5 lesions and/or visceral involvement). Peripheral blood mononuclear cells (PBMCs) were isolated and analyzed via flow cytometry for key immune subsets, including γδ T cells, αβ T cells, and regulatory T cells, with functional assessments performed using cytokine stimulation. Statistical analysis used the Mann-Whitney-Wilcoxon test, with p ≤ 0.05 considered significant.

Results

OMPC patients exhibited significantly increased γδ2+ T cells compared to PMPC, suggesting enhanced immune surveillance in low metastatic burden. A trend toward elevated γδ2+ T cells expressing interferon-gamma (IFN-γ) was observed in PMPC. No significant differences were observed in other immune subsets.

Conclusions

γδ2+ T cells represent a distinct immune subset in PCa, potentially influencing disease progression. Despite the small sample size, these findings highlight γδ2+ T cells as promising biomarkers and therapeutic targets. Prospective studies with a larger sample size are warranted to confirm the significance of these findings and explore the mechanistic roles of these cells and possible clinical applications in metastatic PCa (mPC).

## Introduction

Prostate cancer (PCa) is the most diagnosed malignancy in men in Europe and is the third leading cause of cancer-related mortality in the male population [1].

The disease typically follows a multistep progression, beginning as localized, androgen-sensitive tumors that may eventually evolve into advanced, metastatic castration-resistant PCa, which presents significant therapeutic challenges and poor survival outcomes [2,3]. The five-year overall survival rate for metastatic PCa (mPC) stands at 31% [4].

Oligometastatic PCa (OMPC) is an intermediate state between locally advanced disease and high-volume, metastases, necessitating differentiation in terms of the prognosis and therapeutic strategies [5].

While a universally agreed-upon definition of OMPC and standardized diagnostic criteria have not yet been established, various methodologies are employed in clinical trials. In most studies, the threshold for defining the number of metastases is generally set at fewer than four to six, often involving lymph node (N1 or M1a) or bone (M1b) metastases [6]. OMPC is hypothesized to represent a biologically distinct state that may be amenable to targeted metastasis-directed therapies. In contrast, plurimetastatic PCa (PMPC) involves a higher burden of disease, often including visceral metastases, and typically requires systemic therapeutic approaches [7].

These distinctions are clinically relevant, as multiple studies have demonstrated that the quantity and anatomical distribution of metastatic sites significantly impact survival outcomes [8-10].

Oligometastatic (OM) disease can be further categorized into “synchronous” and “metachronous” subtypes, based on the timing of metastasis relative to the primary cancer diagnosis. Synchronous OM disease refers to the presence of metastatic lesions at the time of initial diagnosis of PCa [11]. In contrast, metachronous (or oligorecurrent) disease is characterized by the development of metastatic lesions at least three months following the treatment of the localized tumor with curative intent [12].

PCa development and progression are driven by factors such as genetic mutations, epigenetic alterations, hormonal dysregulation, and environmental influences [13].

A critical element influencing PCa behavior is the tumor microenvironment (TME), a highly dynamic and complex milieu that encompasses cancer cells, stromal cells, the extracellular matrix, and a wide array of secreted factors such as cytokines, chemokines, and growth factors [14,15].

Unbalanced tumor-infiltrating immune cells within the TME have been shown to promote tumor growth and are associated with poor clinical outcomes in various cancers, including PCa.

PCa is often considered an immunologically “cold” tumor due to its low mutational burden, minimal T-cell infiltration, reduced inflammatory signaling, and the presence of immune checkpoint molecules [16]. Regarding TME, studies have shown that the infiltration of cytotoxic CD8+T and Th1 cells correlates with better outcomes, while elevated levels of regulatory cells (Treg) and tumor-associated macrophages (TAM) promote tumor growth, metastasis, and resistance [17].

A growing body of evidence highlights the role of γδ T cells in antitumor immunity [18]. These cells bridge the gap between innate and adaptive immunity and have demonstrated the ability to mediate rapid responses to infections and malignancies. The Vδ2 T cell receptor (TCR)-expressing (γδ2+) T cells constitute the dominant γδ T cell subset in the peripheral blood. This subset has been shown to display cytotoxic activity and promote immune surveillance against cancer, often through the secretion of interferon-gamma (IFN-γ) [19].

Despite significant advancements in PCa research, the differential immune profiles between OMPC and PMPC remain poorly understood. This study aims to investigate variations in peripheral immune cell populations between OMPC and PMPC, with the goal of identifying immune signatures that could provide insights into the biological mechanisms of metastatic progression. Additionally, by analyzing these differences, the study seeks to inform the development of prognostic biomarkers and targeted therapeutic strategies, particularly in the context of immunotherapy.

## Materials and methods

This retrospective cohort study included patients diagnosed with mPC who received treatment at the Unidade Local de Saúde de Santa Maria between April 2017 and September 2023. Eligible participants were identified based on the following criteria: histologically confirmed prostate adenocarcinoma, evidence of metastatic disease confirmed via imaging (CT scans and bone scintigraphy), and availability of clinical, pathological, and peripheral blood data. Patients were excluded if they had insufficient clinical data, prior immunosuppressive therapy unrelated to PCa, or other concurrent malignancies.

At the time of the initial oncology appointment, patients were stratified into two groups based on metastatic burden. The OMPC group was defined as having ≤5 lesions in either bone or lymph nodes, as assessed by CT and bone scans, consistent with previously published criteria [20-22]. The PMPC group was defined as having >5 lesions or the presence of visceral metastases.

Peripheral blood samples were collected in ethylenediaminetetraacetic acid (EDTA) tubes at enrollment and processed within two hours to isolate peripheral blood mononuclear cells (PBMCs) using the Histopaque-1077 Hybri-Max (Sigma-Aldrich, St. Louis, MO) standard protocol previously published [23,24]. Flow cytometry analysis was performed to quantify immune subsets and functional markers. A standardized antibody panel was used to identify key immune populations, including T cell subsets (divided into γδ T cells (Vδ1 and Vδ2), αβ T cells (CD4 and CD8)), regulatory T cells (Tregs, identified by FOXP3 expression), and effector, memory, and naive phenotypes (defined according to their expression of CD27 and CD45RA). To assess functional activation, PBMCs were stimulated with a cocktail of Brefeldin A (BFA), phorbol 12-myristate 13-acetate (PMA), and ionomycin for three hours prior to flow cytometry analysis, as previously published [23,24]. Cytokines of interest included tumor necrosis factor-alpha (TNF-α), IFN-γ, and interleukin-17 (IL-17). Flow cytometry data were acquired using a BD LSR-Fortessa 2 cytometer (BD Biosciences, Franklin Lakes, NJ), with daily quality control performed using SPHERO™ Rainbow Calibration Particles (Spherotech, Inc., Lake Forest, IL). Gating analysis was conducted using FlowJo™ software (v10.6.2) (FlowJo, LLC, Ashland, OR).

Statistical analysis was performed to compare the median of the cell counts between the OM and plurimetastatic groups. Given the low sample size and the unknown distribution of cell counts, the non-parametric Mann-Whitney U test was used to assess statistical significance. A p-value of <0.05 was considered statistically significant. Due to the exploratory nature of this study, no corrections for multiple comparisons were applied. Statistical analysis was conducted using Python scripts, with data visualization and graphical representation performed using the Matplotlib library (John D. Hunter, Python Software Foundation, Wilmington, DE).

## Results

Baseline characteristics of the study population

A total of 43 patients were included in this study, with a median age at PCa diagnosis of 64 years (range: 50-80). Of these, 17 patients (39.5%) were classified as having OMPC, while 26 (60.5%) patients were categorized as having PMPC (Table 1).

**Table 1:** Baseline characteristics of the study population OMPC: oligometastatic prostate cancer; PMPC: plurimetastatic prostate cancer

	OMPC	PMPC	Total
	17 (39.5%)	26 (60.5%)	43 (100%)
Age at diagnosis, years			
Median (range)	60 (50-80)	66 (50-80)	64 (50-80)
Onset of disease			
De novo mPC	4 (23.5%)	12 (46.2%)	16 (37.2%)
Recurrent mPC	13 (76.5%)	14 (53.8%)	27 (62.8%)
Symptomatic at metastatic disease diagnosis, n (%)			
Yes	2 (11.8%)	9 (34.6%)	11 (25.6%)
Metastatic sites at baseline, n (%)			
Bone	12 (70.6%)	22 (84.6%)	34 (79%)
Lung	0	4 (15.4%)	4 (9.3%)
Lymph nodes	5 (29.4%)	14 (53.8%)	19 (44.1%)
Therapy prior to blood collection			
One line	9 (42.9%)	12 (57.1%)	21 (75%)
Two lines	3 (42.9%)	4 (57.1%)	7 (25%)

Furthermore, 16 patients (37.2%) presented with de novo mPC, predominantly in the PMPC subset (n = 12; 46.2%). 

Symptomatic disease was defined as bone pain, severe fatigue, or weight loss. At the time of metastatic diagnosis, it was observed in 11 patients (25.6%). 

With regard to the location of metastases, bone involvement had roughly the same prevalence in both groups (n = 12, 70.6% in OMPC; n = 22, 84.6% in PMPC), while lymph node involvement was more frequent in the PMPC group (n = 14; 53.8%) compared to the OMPC group (n = 5; 29.4%).

With respect to treatment, 28 patients (65.1%) had started therapy at the time of blood sampling, with a median treatment initiation of five months and 10 days before the blood collection. 

Most patients were undergoing or had completed only one line of therapy for mPC (n = 21; 48.8%). Five patients (17.9%) had received three lines of prior therapy, while two patients (25%) had undergone two lines of previous treatment.

Among patients who had started treatment prior to the blood sampling, 85.7% (n = 24) had received hormone therapy.

Immune cell profile of the study population

The immune cell populations present in the peripheral blood of mPC patients were quantified by flow cytometry.

**Figure 1:**
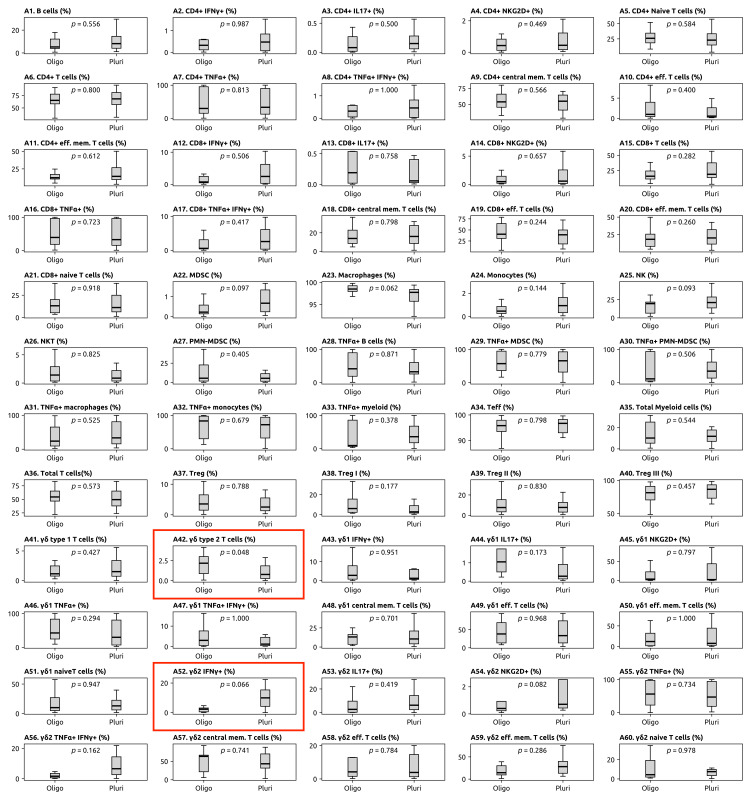
Distribution of immune cell subsets in the peripheral blood of metastatic prostate cancer p-values were determined using the Mann-Whitney-Wilcoxon test, and p ≤ 0.05 was considered significant. MDSC: myeloid-derived suppressor cells; Oligo: oligometastatic; Pluri: plurimetastatic; Teff: effector T; TNF-α: tumor necrosis factor-alpha; Treg: regulatory T cells

An increase in γδ2+ T cells (p = 0.048) was observed in the OMPC group, which was statistically significant for the chosen threshold of p < 0.05. There was a trend toward higher levels of γδ2+ expressing IFN-γ (p = 0.066) in PMPC patients (Table 2). No significant differences were found in other peripheral immune cell populations (Figure 1).

**Table 2:** Immune cell subsets in the peripheral blood of metastatic prostate cancer p-values were determined using the Mann-Whitney-Wilcoxon test, and p ≤ 0.05 was considered significant. OMPC: oligometastatic prostate cancer; PMPC: plurimetastatic prostate cancer

Cell subset	Group	p-value
γδ2+ T cells	OMPC	0.048
γδ2+ expressing IFN-γ	PMPC	0.066

## Discussion

The present study aimed to identify alterations in circulating immune cell subsets among patients with mPC with different metastatic burdens, namely, OMPC and PMPC.

Our findings suggest a significant increase in γδ2+ T cells in the OMPC group, highlighting a possible role for these cells in the immune response within this patient population. γδ2+ T cells exert antitumor effects through production of cytokines like IFN-γ and TNF-α and direct tumor cell lysis. This finding is consistent with prior studies demonstrating the accumulation of γδ2+ T cells in inflammatory and tumor-related contexts, where they can contribute to immune surveillance and antitumor activity [19,25].

While our results align with evidence suggesting that γδ2+ T cells may play a critical role in immune responses against malignancies, they require further validation in larger, more prospective cohorts, particularly those consisting of treatment-naive patients. Moreover, the cross-sectional nature of this study provides only a snapshot of immune cell dynamics, limiting the ability to infer causal relationships between immune alterations, metastatic burden, and prior therapeutic interventions. The influence of previous treatments on immune profiles, which could act as confounding factors, should also be considered in future studies to better understand their impact on immune responses in mPC.

Interestingly, no significant differences were observed in other peripheral immune cell populations across the groups. This could suggest that the observed expansion of γδ2+ T cells is a unique immune feature in OMPC, potentially driven by distinct immune pressures or cytokine milieus associated with these conditions. However, the sample size was relatively small, which may limit the generalizability of our findings. Larger, more diverse cohorts would be essential to confirm these observations and determine the broader applicability of γδ2+ T cells as a biomarker in mPC. The limited sample size also introduces a degree of sampling bias, and larger studies would help mitigate this limitation.

Further research is needed to elucidate the functional role of γδ2+ T cells in OMPC and PMPC. Future studies should investigate the mechanisms underlying their expansion and IFN-γ expression, as well as their interactions with other immune subsets and the TME. Additionally, longitudinal studies would provide valuable insights into how immune cell alterations evolve over time and in response to therapy. Given the potential role of γδ2+ T cells in antitumor immunity, future clinical trials should explore their therapeutic modulation as a strategy to enhance immune responses in mPC.

## Conclusions

Our findings underscore the potential role of γδ2+ T cells as both immune biomarkers and functional mediators in OMPC and PMPC. Their distinct immunological properties suggest a possible influence on disease progression and therapeutic response. However, further studies are required to validate these findings in larger, longitudinal cohorts and to elucidate the underlying mechanisms driving γδ2+ T cell dynamics in mPC. A deeper understanding of these cells may provide novel insights for prognostic stratification and immunotherapeutic strategies in this setting.
